# The Head-Up Tilt Table Test as a Measure of Autonomic Functioning among Patients with Myalgic Encephalomyelitis/Chronic Fatigue Syndrome

**DOI:** 10.3390/jpm14030238

**Published:** 2024-02-23

**Authors:** Leonard A. Jason, William J. McGarrigle, Ruud C. W. Vermeulen

**Affiliations:** 1 Department of Psychology, DePaul University, Chicago, IL 60614, USA; 2 Department of Psychology, University of Kentucky, Lexington, KY 40508, USA; wmcgarrigle@uky.edu; 3 CFS Medical Center, 1114AA Amsterdam, The Netherlands; rv@cvsmemc.nl

**Keywords:** Myalgic Encephalomyelitis/Chronic Fatigue Syndrome, tilt table test, orthostatic intolerance

## Abstract

Individuals with Myalgic Encephalomyelitis/Chronic Fatigue Syndrome (ME/CFS) often experience autonomic symptoms. In the present study, we evaluated 193 adults seeking treatment for ME/CFS, who were recruited from an outpatient clinic. The participants completed a head-up tilt table test to assess two common types of orthostatic intolerance, namely, postural orthostatic tachycardia syndrome (POTS) and orthostatic hypotension (OH). During the tilt test, 32.5% of the participants demonstrated POTS or OH. The participants with either of these two common types of orthostatic intolerance were found to have more problems with sleep and post-exertional malaise as assessed by the DePaul Symptom Questionnaire; these patients also reported more physical and health function limitations. The implications of the findings are discussed.

## 1. Introduction

Patients with Myalgic Encephalomyelitis/Chronic Fatigue Syndrome (ME/CFS) often experience autonomic symptoms, including nausea, headaches, sleep disturbance, and cognitive problems [1]. Patients with ME/CFS also commonly demonstrate elevations in their resting heart rate, systolic blood pressure, and mean arterial blood pressure, and often show a lower stroke index [2]. Up to 75% of adults with ME/CFS have these symptoms, which could be due to a malfunctioning autonomic nervous system [3].

One type of autonomic dysfunction implicated in ME/CFS is orthostatic intolerance (OI), signifying abnormal dynamic blood pressure regulation [4]. OI is defined by an inability to tolerate an upright position and is relieved by rest and recumbence [5]. Common symptoms of OI include dizziness, lightheadedness, and syncope, among others. One type of OI in patients with ME/CFS is vasovagal syncope (also called simple fainting or neurocardiogenic or neurally mediated syncope). Two other common subtypes are postural orthostatic tachycardia syndrome (POTS) and orthostatic hypotension (OH). POTS is marked by a substantial increase in the heart rate when transitioning from the supine to an upright position [6], whereas OH involves a fall in blood pressure upon standing [7]. These conditions contribute to ME/CFS symptoms broadly and are associated with a decrease in the quality of life [8]. Schultz, Katz, Bockian, and Jason [9] found significant correlations between youth self-reported levels of orthostatic and autonomic functioning and physician measurement of orthostatic functioning; however, that study did not involve a head-up tilt table test, which serves as an autonomic assessment of OI. 

A head-up tilt table test can assess these two common types of OI—POTS and OH. The head-up tilt table test is one of the major assessment tools that has been used in ME/CFS research trials. Another stressor assessment tool that has been used to assess physiological systems involving ME/CFS is a two-day cardiopulmonary exercise test (CPET) performed 24 h apart [10]. In patients with ME/CFS, the CPET demonstrates an inability to reproduce maximal or anaerobic threshold measures on the second day; values on the second CPET are much lower than those on the first CPET. However, this test may induce severe exacerbation of symptoms in these patients. Due to this, several researchers [11] have suggested using a single-day CPET, but a single day does not capture the aerobic impairment. Keller, Pryor, and Giloteaux [11] found that a single CPET resulted in the classification of 12 of 22 patients with ME/CFS as having little or no impairment, and 8 patients as having mild/moderate impairment. But data from the second day’s CPET indicated that aerobic energy-producing processes failed to respond normally to exercise. As Batemen et al. [10] suggest, the CPET should be used for disability testing only, as these tests involve a stressor that may induce severe or long-lasting post-exertional malaise.

The present study involved a sample of ME/CFS patients from the Netherlands. We explored the percentage of patients who had OI (using either tilt-table testing or self-reports) so that we could determine how common OI is among patients with ME/CFS. We were interested in exploring whether OI has a high prevalence among patients with ME/CFS, such as post-exertional malaise, cognitive impairment, and unrefreshing sleep. If patients with ME/CFS are selected from tertiary care settings that specialize in OI, it is more likely that OI would be a prevalent symptom of ME/CFS, but they might occur less frequently in non-tertiary care settings. In other words, differences could be due to where the patients are recruited, as specialty clinics tend to attract more severely impaired patients [3]. The present study hypothesized that patients recruited from a setting that did not specialize in OI care might have lower rates of OI. The following study used the head-up tilt table test to assess POTS and OH among an adult sample of patients with ME/CFS, and the outcomes of this test were related to self-reported symptoms and overall functioning.

## 2. Method

### 2.1. Participants

The sample consisted of 193 adults with a physician report of ME/CFS and referred to an outpatient clinic in the Netherlands (the CFS Medical Center in Amsterdam).

### 2.2. Tilt Table Test Procedure

The head-up tilt table test [12] served as an autonomic assessment of orthostatic intolerance. During the test, which lasted 20 min, an appropriately sized cuff was placed on the participant’s upper arm and the participant was instructed to remain still and silent in the supine position for 10 min. At that time, blood pressure and pulse were measured with a sphygmomanometer (Omron M6). After 10 min, the table was raised to a 70-degree head-up tilt for another 10 min. The test was terminated after 10 min in the tilted position or sooner if the participant reported complaints indicating insufficient cerebral perfusion.

The participants were labeled as positive for orthostatic intolerance (OI+) if they demonstrated either POTS or OH during the head-up tilt table test. OI− indicated that the patient did not have POTS or OH. POTS was defined as a heart rate increase of ≥30 bpm that is sustained (i.e., lasting at least two consecutive minutes) within 10 min of the tilt; OH was defined as a sustained decrease of at least 20 mmHg in systolic blood pressure or 10 mm Hg of diastolic blood pressure within 3 min of the tilt [13]. Baseline blood pressure and pulse were defined by those collected at the ninth minute of the test, just before the tilt.

### 2.3. Measures

#### 2.3.1. The DePaul Symptom Questionnaire

The DePaul Symptom Questionnaire (DSQ-1) was completed by the participants. The DSQ-1 is a 54-item self-report that measures ME/CFS symptomology, demographics, and medical, occupational, and social history [14]. Using a 5-point Likert scale, the DSQ-1 indexes the frequency and severity of symptoms within the past 6 months. The scale for frequency is as follows: 0 = *none of the time*, 1 = *a little of the time*, 2 = *about half of the time*, 3 = *most of the time*, and 4 = *all of the time*. The scale for severity is as follows: 0 = symptom not present, 1 = *mild*, 2 = *moderate*, 3 = *severe*, 4 = *very severe*. The scores are converted into a 100-point scale, and the frequency and severity scores of each symptom are averaged into one composite score of the symptom.

The DSQ-1 has shown good to excellent test–retest reliability for those with ME/CFS and individuals within the control groups [15]. Factor analytic studies using this instrument have identified cardinal symptom clusters, or core domains, of ME/CFS [16]. The DSQ-1 has been used to differentiate ME/CFS from other chronic illnesses, like multiple sclerosis [17]. The Shared Library of Research Electronic Data Capture (REDCap) offers access to the DSQ-1 through the host of DePaul University.

#### 2.3.2. The 36-Item Short-Form Health Survey (SF-36)

The participants in this study also completed the 36-item Short-Form Health Survey (SF-36). The SF-36 is a self-report inventory that focuses on eight different domains: Physical Functioning, Role Physical, Bodily Pain, General Health Perceptions, Vitality, Social Functioning, Role Emotional, and Mental Health [18]. Items are rated on a five-point Likert scale, with higher scores indicating better health, or that a patient’s functioning is being less impacted by their health. The SF-36 is considered a reliable and valid instrument capable of differentiating between patient and non-patient populations [19].

## 3. Results

### 3.1. Demographics

The age ranged from 18 to 68 years (*M* = 38.3, *SD* = 12.16). Most participants were female (78.8%). A duration of illness longer than two years was reported by 67.9% of the sample. Regarding work status, patients were categorized as either Working Part- or Full-Time/Students (52.8%) or Disabled/Unemployed/Retired (47.2%). There were no significant differences observed between the OI+ and OI− groups on demographic characteristics.

### 3.2. Outcomes

During the tilt table test, 32.5% (*n* = 63) of the participants demonstrated orthostatic intolerance (POTS or OH). Table 1 provides the differences between the OI+ and OI− groups for the main DSQ-1 domains and symptoms. On average, OI− group had significantly lower scores (i.e., less frequent and severe) on the Sleep and Post-Exertional Malaise symptom domain and the following symptom items: unrefreshing sleep, difficulty falling asleep, difficulty staying asleep, waking up early, trouble forming words, and feeling chills or shivers. As displayed in Table 2, the OI+ group had significantly greater impairment in the SF-36 Physical Functioning and General Health domains.

## 4. Discussion

This study’s major finding is that, on average, patients with POTS or OH experienced more symptoms and functional limitations than those not experiencing POTS or OH. Interestingly, only 32.5% of the participants demonstrated OI (POTS or OH) during the tilt table test. Although it is not surprising that those with POTS or OH have more physical and health functional problems, we expected to find a higher percentage of individuals with POTS or OH. It is possible that the low rate of 32.5% demonstrating POTS or OH could be due to not including other forms of OI, such as vasovagal syncope. It is also possible that cerebral blood flow is reduced in ME/CFS during head-up tilt testing even in the absence of hypotension or tachycardia [20]. It is also plausible that given the high percentage of patients with POTS or OH that were either working or in the student status, this sample had relatively less impairment. If this is the case, we speculate that the head-up tilt test may be more effective at detecting OI among more severely impaired patient cohorts, such as those within tertiary care settings.

In addition to functional limitations, we found that patients with POTS or OH reported elevated scores on the DSQ-1 post-exertional malaise and neurocognitive symptom domains; this group also demonstrated higher composite scores on individual DSQ-1 items, including unrefreshing sleep, difficulty falling asleep, difficulty staying asleep, waking up early, trouble forming words, and feeling chills or shivers. The wide assortment of sleep, neurocognitive, and neuroendocrine features suggests that those with POTS or OH have symptoms that are likely contributing to their functional limitations.

Notably, among our OI+ group, only 37.7% indicated “Feeling unsteady on your feet, like you might fall”, using the threshold score of at least moderate severity and at least half the time. Comparable results were found for the item “Dizziness or fainting”, where only 32.3% of those with a positive tilt test met the threshold burden of at least moderate severity and frequency of half the time or more. These findings suggest that the majority of individuals in our sample with positive tilt table test data did not meet the threshold for these OI self-report items being a burden to the patients.

The findings from this study have relevance to the Institute of Medicine [21] report that provided a new case definition for ME/CFS. In brief, the new clinical case definition required a substantial reduction in pre-illness levels of activity, post-exertional malaise, unrefreshing sleep, and either neurocognitive impairment and/or OI. The IOM report also operationalized OI as having a moderate or greater frequency and severity of symptoms. These new criteria had some similarities with prior ME/CFS case definitions and their stipulation of symptoms [22,23], but the IOM criteria was the first time an ME/CFS case definition required either neurocognitive impairment and/or OI [24].

Focusing on the IOM case definition, Jason et al. [24] found that 67% of patients with ME/CFS report OI, whereas 93% report cognitive impairment. These researchers found that by using the OI symptoms instead of neurocognitive impairment, only 2% more participants met the IOM criteria than if the criteria had only required cognitive impairment. A different approach was tried by Chu et al. [25], but her team utilized a categorical response of “yes” and “no” to measure “feeling sick, uncomfortable, or fainting while standing.” In contrast to Jason et al.’s [24] findings, Chu et al. (2017) found that 92% of participants reported OI and 87% of participants endorsed cognitive impairment. Chu et al.’s group operationalization of OI allowed for 13% more participants to meet the IOM criteria than if participants were required to endorse cognitive impairment alone. Chu and colleagues hypothesized the discrepancy in findings from Jason et al. [24] might have been due to the researchers’ use of “less common symptoms” to represent OI (e.g., shortness of breath and irregular heartbeats). Additionally, Chu and colleagues did not require minimum frequency and severity thresholds as required by the IOM.

To deal with this controversy, Gaglio et al. [26] assessed different methods of operationalizing OI for the IOM criteria. With a sample of two-hundred and forty-two participants who completed the DSQ, they examined how many participants met the IOM criteria while endorsing different frequencies and severities of various OI symptoms. While neurocognitive impairment occurred in 93.4% of patients with ME/CFS, OI without concurrent neurocognitive symptoms only allowed for an additional 1.7–4.5% of participants to meet the IOM criteria. These results do not support the IOM’s inclusion of neurocognitive impairment and OI as interchangeable symptoms.

Although as indicated in the introduction, OI can result in significant impairment, it has not been found to be among the most prevalent ME/CFS symptoms [24]. Other researchers have found similar results, such as Schondorf et al.’s [27] study where only 40% of their ME/CFS sample had a positive tilt test (indicative of OI). In addition, Timmers et al. [28] reported an even lower percentage of 27.8%. In addition, LaManca et al. [29] were not able to find any significant differences in presyncope symptoms or heart rate and blood pressure changes (indicative of OI assessment) between those with ME/CFS and controls. These studies along with the present study indicate that OI might not be considered a core symptom of ME/CFS.

Still, OI is a symptom of at least some patients with ME/CFS. In those patients with OI, there appears to be either too little or too much expression of insufficient control of the autonomic systems. In the upright position, the pressure in the circulation in the lower part of the body increases and the response is an increase in the tension of the vessel walls. Too little and the blood pools in the lower part and too much increases the resistance, expressed as an increase in the diastolic blood pressure, a lower pulse pressure, and a lower stroke volume. The increase in the heart rate is an attempt to compensate for the lower cardiac output. In ME/CFS, there is also a complicating low blood volume, sometimes comparable to a hypovolemic shock in which lifting of the head results in a major increase in the heart rate. There probably is some interference and bias between symptoms and results of the table test.

There are many other potential biological ways to identify the multiple causes of OI symptoms, including anemia (which can be determined by routine blood tests, as low normal hemoglobin may affect oxygen supply to the brain) [30], oxygen dissociation (in a person with normal hemoglobin and hematocrit, the red blood cells may have a strong affinity for holding onto the oxygen) [31], Ehlers–Danlos syndrome (where the lax blood vessels in lower extremities allow blood to pool and blood does not reach the heart and brain adequately upon standing) [32], vasopressin/ADH deficiency (diabetes insipidus) [33], and low blood volume (which could be related to aldosterone levels) [34] (Jacob et al., 1998). Ryabkova et al. [35] found similar patterns of dysautonomia involving heart rate variability, blood pressure variability, and baroreflex failure in patients with ME/CFS and Long COVID. After the head-up tilt test, Swai et al. [36] found that patients with POTS have lower heart rate variability in terms of time domain measure but not in terms of frequency domain measure. In addition, a subgroup of ME patients have autoantibodies to adrenergic receptors in the central nervous system [37] and this is probably related to OI. Certainly, OI is complex and multiple methods might need to be employed to adequately assess and understand these symptoms.

Physiological testing such as tilt table testing and exercise testing (including VO2 max) have been used to address specific questions, often in consultation with a specialist [38]. Tilting and exercise have been used as a provocation in ME/CFS specifically because they provoke the disease symptoms, thus making it easier to see abnormalities in metabolism, skeletal muscle, gene expression, neurological and cognitive measures, cardiovascular/autonomic reflex abnormalities, immune abnormalities, and oxidative stress and alterations in the microbiome. Keller et al. [11] demonstrate that patients with ME/CFS have a different response to CPET testing and the present study suggests that at least some patients with ME/CFS exhibit OI, but not at the same percentage as other classic ME/CFS symptoms such as post-exertional malaise and cognitive impairment.

This study had several limitations. For example, we did not assess OI symptoms following the tilt table test; follow-up data might have allowed us to better evaluate the effects of this stressor on the patients. In addition, the sample size for the OI+ group was considerably smaller than that of the OI− group. Future studies would benefit from more extensive monitoring of autonomic symptom indicators using larger cohorts of patients with and without OI.

In conclusion, our study found evidence that those with POTS or OH have more limitations as well as symptoms than those without POTS or OH. Even so, only about one-third of the patients had POTS or OH based on the tilt table test. Further research is needed to determine the relationship between positive tilt test data and self-reported symptoms of OI, given that the Institute of Medicine [21] currently lists OI and/or cognitive impairment as one of the defining symptoms of ME/CFS and the present study suggests that OI might be an important feature of ME/CFS but not a core symptom that is essential to the syndrome (i.e., post-exertional malaise, unrefreshing sleep, and cognitive impairment).

## Figures and Tables

**Table 1:** Significant Domain and Symptom Differences.

	OI− (*n* = 130) *M* (SD)	OI+ (*n* = 63) *M* (SD)	*p*
Sleep Domain	50.83 (17.24)	43.71 (17.95)	0.01
Unrefreshed	86.29 (14.16)	79.75 (12.24)	0.01
Difficulty falling asleep	55.11 (29.39)	42.36 (27.55)	0.00
Difficulty staying asleep	53.50 (32.05)	43.06 (30.97)	0.02
Waking up early	40.46 (32.99)	32.29 (30.72)	0.07
PEM Domain	72.90 (17.19)	64.79 (19.26)	0.00
Trouble forming words	58.20 (23.78)	48.50 (22.13)	0.00
Feeling chills or shivers	31.85 (24.42)	21.53 (22.21)	0.00

**Table 2:** Comparison of SF-36 domain composite scores.

	OI− (*n* = 130) *M* (SD)	OI+ (*n* = 63) *M* (SD)	*p*
Physical Functioning	43.40 (23.46)	36.11 (23.95)	0.04
Role Physical	5.81 (19.64)	3.57 (14.80)	0.42
Bodily Pain	44.81 (24.88)	41.11 (26.40)	0.34
General Health	28.53 (18.08)	20.06 (14.80)	0.00
Vitality	23.92 (15.36)	19.76 (13.18)	0.07
Role Emotional	68.22 (42.63)	66.67 (42.75)	0.81
Mental Health	61.88 (16.65)	60.00 (17.88)	0.48

## Data Availability

Data will be made available by contacting the first author.
